# Exploring school nurses’ potential to strengthen young people’s resilience to misinformation by promoting critical health literacy in Norway

**DOI:** 10.1093/heapro/daag083

**Published:** 2026-06-26

**Authors:** Christine Holst, Heather Menzies Munthe-Kaas, Lena Victoria Nordheim, Mariann Enoksen, Sarah Rosenbaum

**Affiliations:** Centre for Epidemic Interventions Research, Norwegian Institute of Public Health, P.O.Box 222 Skøyen, 0213 Oslo, Norway; Centre for Epidemic Interventions Research, Norwegian Institute of Public Health, P.O.Box 222 Skøyen, 0213 Oslo, Norway; Western Norway University of Applied Sciences, P.O.Box 7030, 5020 Bergen, Norway; Western Norway University of Applied Sciences, P.O.Box 7030, 5020 Bergen, Norway; Centre for Epidemic Interventions Research, Norwegian Institute of Public Health, P.O.Box 222 Skøyen, 0213 Oslo, Norway

**Keywords:** critical health literacy, critical thinking, health literacy, health education, misinformation, school nurses, public health nurses, educational resource design

## Abstract

Young people are increasingly exposed to health misinformation, especially in social media. They often lack the skills necessary to think critically about misleading claims they encounter and decisions they make. The Informed Health Choices framework provides principles for critical health literacy. These can be taught using resources shown to have positive impact on student’s abilities to think critically about health claims and make informed decisions. We explored the potential for school nurses in Norway to use a tailored set of teaching resources to support young people’s resilience to health misinformation harm. We conducted this qualitative context analysis for the purpose of informing resource development. We identified key issues related to school nurses’ capability, opportunity, and motivation to teach a set of critical health literacy principles and their needs regarding resource design. We conducted interviews, a focus group discussion, and co-design workshop and applied framework analysis to synthesize findings. School nurses in Norway seem keenly aware of young people’s vulnerability to harms posed by misinformation and likely motivated to expand their teaching role to include teaching critical health literacy. However, critical health literacy knowledge varies among school nurses. Teaching opportunities are limited, largely due to many mandated tasks. Therefore, content needs to be clearly aligned with their nurses’ existing responsibilities and school curricula, and resources should be designed to support school nurses with limited time and critical health literacy skills, adaptable to emerging topics, be engaging to students, and facilitate school collaboration.

Contribution to Health Promotion

- Health misinformation is a societal threat. Schools are an important arena for equitably strengthening critical health literacy but lack explicit curricular goals.
- School nurses in Norway witness students’ vulnerability to health misinformation first hand, making them likely motivated to teach critical health literacy.
- Their teaching opportunities are scarce, so resources must be flexible and aligned to schools’ goals and school nurses’ guidelines.
- Teaching resources should not require advanced critical health literacy skills.
- School collaboration is key. This should not be left up to individual school nurses but pursued across sectors by regional or national policymakers.

## Introduction

Misinformation—misleading, inaccurate, or false information spread regardless of intent—is a growing public health threat (Do Nascimento *et al*. 2022, Kbaier *et al*. 2024). It is prevalent on social media, spreading faster than accurate information, especially when it has novel or emotional appeal (Vosoughi *et al*. 2018). Reduced fact-checking policies create unvetted environments where misinformation thrives (Zhou 2025). Adolescents, who rely heavily on social media for information, are particularly vulnerable (Newman *et al*. 2019).

Beliefs in misinformation or misleading claims about the effects of health interventions can cause harm, by delaying or preventing effective care, impacting mental health, leading to unnecessary spending, and exacerbating public health crises (Do Nascimento *et al*. 2022, Wilson *et al*. 2023). Studies show that both students and adults in Norway lack basic skills to assess the reliability of claims about the effect of treatments or other health-related interventions (Nordheim *et al*. 2019, Dahlgren *et al*. 2021). In practice, this can have serious consequences, such as reduced use of birth control pills in favour of natural approaches to contraception, leading to an increase in adolescent pregnancy (NRK 2024). A public health approach to misinformation should include strengthening the resilience of individuals and communities through education (Denniss and Lindberg 2025). This view is supported by Scherer *et al*. (2021) who found that low health literacy is a predictor for susceptibility to online health misinformation

One solution is supporting people from a young age to identify health claims, consider their reliability, and develop their critical thinking competency and decision-making skills. To address this, an international network funded by the Norwegian Research Council developed the Informed Health Choices (IHC) Key Concepts framework (Austvoll-Dahlgren *et al*. 2015, Chalmers *et al*. 2018, Oxman *et al*. 2019, Oxman *et al*. 2022): a comprehensive set of 49 concepts that can be selected for designing tailored curricula, educational resources, and evaluation tools (Austvoll-Dahlgren *et al*. 2017). The concepts are grouped into three categories: thinking critically about health intervention claims and their basis, thinking critically about fair comparisons (studies measuring effect), and making informed decisions (Supplementary File S1). These concepts can be applied to both individual and group decision making and are applicable to assessing claims about interventions also in other areas, e.g. education or crime prevention (Aronson *et al*. 2019). Learning resources have, to date, focused on facilitating student understanding of some of these concepts in moderated learning environments, such as the classroom (Oxman *et al*. 2016, Rosenbaum *et al*. 2024). The aim of the resources has been knowledge acquisition—teaching students what the concepts are (definitions and explanations)—and skill acquisition, providing examples and opportunities of how to apply their knowledge to various settings (e.g. identifying health claims and considering whether the bases for claims are strong or weak).

Within moderated contexts, such as schools, teachers have the opportunity to support students in acquiring and applying new knowledge. For the purpose of this study, and within the context of the IHC framework, knowledge is necessary for developing skills related to critical health literacy—specifically assessing claims, thinking critically about evidence, and making informed decisions, but it is not sufficient. In order to cultivate critical health literacy (as defined in this study as the ability to recognize claims, interrogate the basis for the claim, and make informed decisions), one must also develop a set of competencies and dispositions that are acquired and strengthened through exposure, practice, and application in multiple and various settings.

In a comparison with 22 other frameworks related to critical thinking, IHC Key Concepts were shown to be closely aligned with broader concepts of ‘health literacy’ and ‘critical health literacy’ (Oxman and García 2020). For definitions of critical thinking (Ennis and Ennis 2003–2024), health literacy (Sørensen *et al*. 2012), critical health literacy (Nutbeam 2008, Smith *et al*. 2013), and IHC Key Concepts (Austvoll-Dahlgren *et al*. 2015), see Table 1.

**Table 1:** Definitions.

Critical thinking	Reasonable reflective thinking focused on deciding what to believe or do (Ennis and Ennis 2003–2024)
Health literacy	People’s knowledge, motivation, and competences to access, understand, appraise, and apply health information to make judgements and take decisions in everyday life concerning healthcare, disease prevention, and health promotion to maintain or improve quality of life during the life course (Sørensen *et al*., 2012)
Critical health literacy	Higher-level cognitive and social skills to critically analyse information and use information to exert greater control over situations (Nutbeam 2008, Smith *et al*. 2013)
IHC Key Concepts	A framework of principles for thinking critically and making informed decisions about things people do to maintain or improve their health, to be used for designing curricula, learning resources or evaluation tools (Austvoll-Dahlgren *et al*., 2015)

In this article, we will use the term ‘critical health literacy’ to mean an individual’s ability to understand and apply IHC Key Concepts while acknowledging that the term may be used differently elsewhere (Chinn 2011). Examples of concepts include recognizing unreliable claims about treatment effects (e.g. claims based on anecdotes), recognizing characteristics of fair comparisons (e.g. studies that have control groups), and understanding how individuals may make different decisions based on the same evidence.

Teams have previously developed and evaluated teaching resources based on selected Key Concepts (Nsangi *et al*. 2017, 2020b, Chesire *et al*. 2023, Rosenbaum *et al*. 2024). In large studies in East Africa, resources have demonstrated an effect on ability to understand and apply concepts for at least 1 year among students aged 10–12 and 13–14 (Nsangi *et al*. 2020a, Chesire *et al*. 2024a). They have been translated and piloted in many countries, including Norway. However, research indicates that inclusion in national curricula is paramount to implementation (Nsangi *et al*. 2019, Mugisha *et al*. 2024, Ssenyonga *et al*. 2024, Chesire *et al*. 2024b). Since critical health literacy is not directly included as an explicit learning goal in the Norwegian K-12 curriculum, we sought to explore the school health services as a potential pathway to teaching students.

### School health services and critical health literacy in Norway

According to the Norwegian Directorate of Health, the school health service aims to promote students’ health, well-being, learning, and health literacy (https://www.helsenorge.no/en/help-services-in-the-municipalities/school-health-service/). All primary and secondary schools have part- or full-time nurses provided by school health services, responsible for health consultations, screenings, vaccinations, teaching, parent meetings, and school collaboration. There is a growing body of research in Norway on the intersection between school health services and children’s health literacy, including surveys, measurement development, interventions for mental health literacy, and physical education’s role in fostering critical health literacy (Haugen *et al*. 2023, Riiser *et al*. 2024). In a literature review of studies prior to 2020, authors found that interdisciplinary collaboration between schools and the school health services in Norway often involved initiatives related to mental health or targeted students with specific vulnerabilities, such as students who experienced bullying. There were very few studies describing universal, health-promoting collaborations, and none that involved teaching critical health literacy (Pedersen and Wollscheid 2024).

#### Aim and objectives

The aim of this study is to explore Norwegian school health nurses’ potential for teaching a set of IHC Key Concepts to strengthen students’ critical thinking and resilience to health misinformation, for the purposes of informing subsequent design of teaching resources. Assuming school nurses do not currently teach IHC Key Concepts, such teaching would entail a behaviour change. Therefore, we have organized our objectives according to the top level themes in Michie’s behaviour change model (COM-B) (Michie *et al*. 2011) (capability, opportunity, and motivation). Although primarily used in implementation research, the COM-B model is useful for exploring behaviour change factors relevant to intervention design (Rosenbaum *et al*. 2024).

Study objectives:

- To identify key issues related to school nurses’ capability, opportunity, and motivation to teaching IHC Key Concepts, for the purposes of informing the design of teaching resources that can help strengthen critical health literacy as a means of eventually building student resilience to health misinformation
- To identify school nurses needs related to teaching resources

## Materials and methods

**Figure 1:**
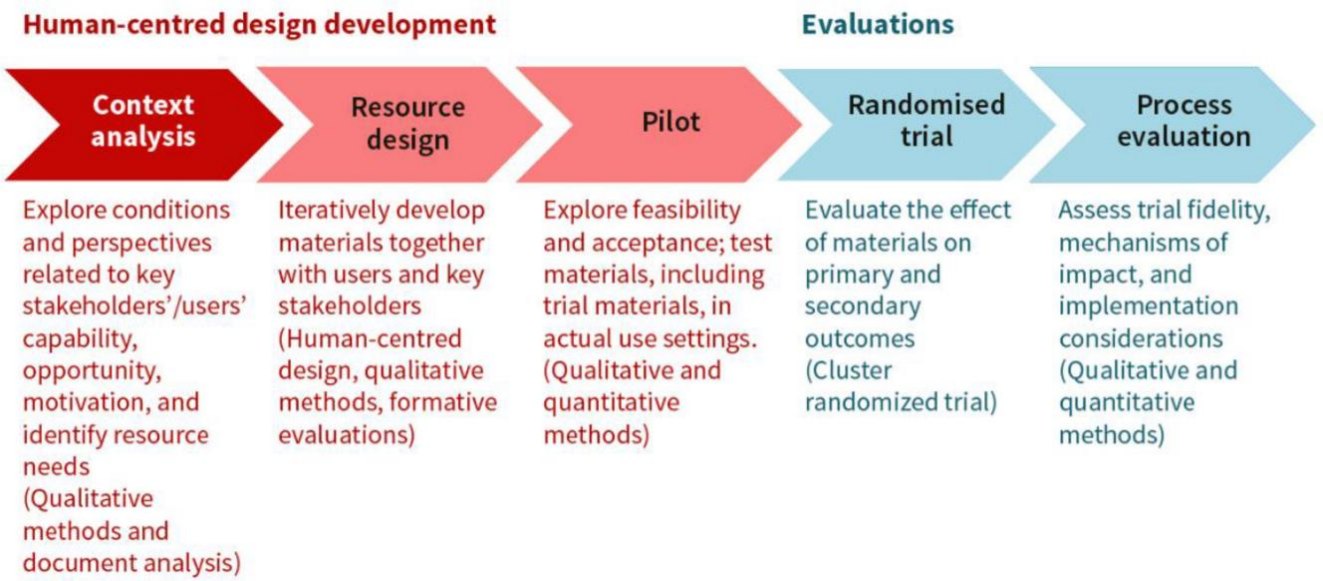
Intervention development and evaluation.

This study employs qualitative methods in a context analysis and the first of five planned studies to develop and evaluate teaching resources for school nurses (see Fig. 1). Our inquiry is grounded in a human-centred design (HCD) approach to intervention development. HCD is an iterative, collaborative process of innovation centred around users’ and key stakeholders’ needs and their context (Göttgens and Oertelt-Prigione 2021). The context analysis is thus a critical first step in understanding the setting in which an intervention will be developed, including identifying existing practice and guidelines, needs among relevant stakeholders, and facilitators and barriers to implementing the target intervention.

To explore school nurses’ conditions and perspectives related to teaching critical health literacy, we organized semistructured interviews, a focus group discussion (FGD), and a co-design workshop. We used the Consolidated criteria for reporting qualitative research (COREQ) (Supplementary File S2) (Tong *et al*. 2007).

In parallel, we conducted two document analyses: (i) examining regulatory and guidance documents for school health services in Norway to assess whether teaching critical health literacy overlaps with existing obligations and (ii) higher education curricula for school nurses to assess whether these students are taught critical health literacy skills. However, due to the narrow contextual relevance (Norway only), we did not include these when writing up this study. See Supplementary File S3 for summaries of these analyses.

### Participant selection and recruitment

We used a combination of snowballing, purposive, and convenience sampling to identify and recruit school nurses, teachers, and other relevant stakeholders employed in government, research, and schools (Table 2). We sought diversity among participants in terms of geography and experience with students of varying ages.

**Table 2:** Selection, recruitment, and compensation.

	Interviews	Focus group and workshop
Participants	Key informants within government, research, and professional organizations; school nurses; teachers	School nurses employed at elementary, lower, and upper secondary schools
Selection	Snowballing, purposive, and convenience sampling, starting with a professional organization	Convenience sampling via personal and professional contacts; direct outreach to schools
Recruitment	Email and phone invitations	Email and phone invitations
Existing relationships	Two employed at the same institution as three authors but never met One in personal network	One in personal network
Compensation	None	Meals and refreshments, reimbursement for travel, hourly compensation offered

### Data collection

#### Semistructured interviews

At least two researchers trained in qualitative research methods conducted individual interviews (≤60 min) via Microsoft Teams using a semistructured guide. One researcher led the interview, while the other observed, took notes, and asked follow-up and clarification questions. We posed questions about participants’ views related to critical thinking and critical health literacy among young people, school nurses’ current roles and teaching opportunities, and characteristics of teaching resources they considered useful. We used Microsoft Teams to record/transcribe all but one interview where we only took notes. We anonymized and securely stored transcriptions in a secure zone of the NIPH server.

#### Focus group discussion

We conducted a 2-h face-to-face FGD in June 2023 at our institute with five school nurses. The venue was accessible by elevator. The three researchers present were trained in qualitative methods; one facilitated using a semistructured guide, while two observed, took notes, and asked additional questions. We briefly presented the aim of the study and then asked participants to describe and discuss their current roles and work contexts, such as tasks, experiences teaching in classrooms, types of interactions with students, and challenges, as well as views about misinformation. We used Microsoft Teams for audio recording/transcription and took notes.

#### Co-design workshop

A full-day workshop in September 2023 involved the same FGD participants at the same venue. Workshop goals were to (i) prioritize which age groups to pilot initial resources with and which IHC Key Concepts were most important for these groups and (ii) create rapid prototypes based on ideas for resources. Participants voted to select Key Concepts and worked in pairs to sketch and present teaching resource ideas. One researcher facilitated while two assisted. The session was not recorded, but researchers consolidated notes afterwards.

### Data analysis

#### Interviews and FGDs

Our analysis was structured around the three top-level categories of the COM-B behaviour change framework, capability, opportunity, and motivation, and informed by thematic findings from similar studies (Mugisha *et al*. 2021, Chesire *et al*. 2022, Ssenyonga *et al*. 2022). Under ‘capability’, we explored aspects related to the school nurse role and existing practice, their competence, and characteristics of new resources considered desirable for supporting critical health literacy teaching. For ‘opportunity’, we examined the contexts and conditions that enable or constrain teaching, including available teaching opportunities and meeting points, capacity and time, collaboration with teachers and other stakeholders, relevant health topics, and how decisions about content are made within their tasks. Finally, under ‘motivation’, we considered factors influencing willingness and perceived need to teach critical health literacy, the demand for school nurse-led teaching, and the role of existing resources in shaping motivation.

Three researchers conducted the analysis. We familiarized ourselves with all the transcripts. One researcher coded the transcriptions using the COM-B framework and added additional subthemes or codes when necessary. A second researcher reviewed each transcript and added codes or made suggestions for different codes as necessary. All three researchers discussed in plenum all codes (themes and subthemes), and any disagreements (e.g. if data belonged to a opportunity category or a motivation category) were discussed until consensus was reached.

See Supplementary File S4 for both the initial and final coding schemes.

#### Co-design workshop

We consolidated and analysed observational notes in relation to interview and FGD data. Unlike the other data sources, we did not analyse workshop insights using the framework described above but rather analysed under the subtheme ‘New Resources: Desirable characteristics and ideas’.

### Reflexivity

Research team members are all female. Three have extensive experience developing and researching public health interventions. One has expertise from the school health services. One was a member of the team that developed the IHC Key Concepts framework. All have personal experience as parents or students with the Norwegian school system and school nurses. The team strongly believes in the importance of critical health literacy, and this may have impacted our interactions with participants and interpretations. We took steps to counteract this, described in the Discussion section.

### Use of artificial intelligence

We used ChatGPT to help shorten sections of text in the final draft of the manuscript.

## Ethical approval

We consulted the Data Protection Officer at the Norwegian Institute of Public Health (NIPH) who approved the project’s privacy provisions in our protocol (11.8.2023). Submission was not required to Regional Committees for Medical and Health Research Ethics (application number 644961).

All participants in the interviews and focus groups received information in advance, including the study purpose, data management plans, and their rights as participants (e.g. right to withdraw). We gathered informed consent for participation and consent for recording separately.

## Results

We recruited five school nurses to participate in the FGD and workshop and interviewed seven people (six with school health service background) (Table 3). Participants came from Akershus, Buskerud, Innlandet, Oslo, and Trøndelag counties in Norway.

**Table 3:** Participant characteristics.

#	Participation	Gender	Profession	School level/employer type	Relevant work experience
Person1	FGD, workshop	Female	School nurse	Elementary	1 year
Person2	FGD, workshop	Female	School nurse	Elementary	10 years
Person3	FGD, workshop	Female	School nurse	Lower secondary	>20 years
Person4	FGD, workshop	Female	School nurse	Upper secondary	>20 years
Person5	FGD, workshop	Female	School nurse	Upper secondary	10 years
Person6	Interview	Female	Researcher/doctor	Research institution	>20 years
Person7	Interview	Female	School nurse/researcher	Research institution	10 years
Person8	Interview	Female	School nurse	Professional organization	>20 years
Person9	Interview	Female	School nurse/project manager	Local government	>20 years
Person10	Interview	Female	Teacher	Local government	10 years
Person11	Interview	Female	School nurse/consultant	National government	Not reported
Person12	Interview	Female	School nurse/consultant	National government	Not reported

Findings are organized under the Motivation, Capability, Opportunity, and Findings related to resource development sections. We present the findings in this order because insights we gathered regarding capability and opportunity need to be considered in light of the themes we identified related to motivation. All data contributed ultimately to the development of the resources, and the themes below are developed using all collected data. Participant quotes are specified as coming from interview, FGD, or workshop data.

### Motivation

Norwegian school nurses are likely to be motivated to teach critical health literacy because they understand the consequences of believing unreliable health misinformation and witness students’ broad exposure to it. Promoting health literacy is already a part of their professional identity, but they may feel that teaching critical health literacy conflicts with their other duties. Some may feel that the responsibility for teaching this type of critical thinking should lie with schools instead.

#### School nurses have first-hand experience of the seriousness of the problem

Participants in this study were acutely aware of young people’s vulnerability to health misinformation, and all agreed that critical thinking about health is a crucial skill for young people to avoid poor choices:

Young people are bombarded with health information through social media, even at school, so I think it is absolutely fundamental that [we] work to increase that knowledge…. (interview, researcher/school nurse)

Participants working in schools reported a constant pressure to deal with students’ exposure to ever-changing topics of misinformation trending on social media. Current examples circulating among students were increased self-diagnoses of Attention deficit hyperactivity disorder (ADHD) and growing scepticism about hormonal prevention. A teaching resource that helped them communicate about emerging topics would be useful:

No matter what we're talking about, we can have this [critical health literacy] as a backdrop. (interview, researcher/school nurse)

They emphasized promoting critical health literacy skills early, ideally before age 10, when many have their own smartphones. Some even suggested kindergarten:

It must be age-appropriate, of course, but as early as possible, I think. (FGD, school nurse)

FGD participants pointed out that some young people experience violence in close relationships, substance abuse, and mental disorders and that for those students (and their school nurses), critical health literacy might not be the most pressing priority. Nevertheless, they discussed that learning to reflect on the trustworthiness of health claims could be useful for dealing with other issues and be a part of a larger strategy aimed at setting boundaries.

#### Promoting health literacy is central to school nurse identity but conflicts with other demands

Participants spoke synonymously of critical health literacy and health literacy. They expressed that promoting health literacy was already a part of their job description to carry out preventive work:

…. Developing students’ health literacy is something that is already being done—out there in service…. (interview, government consultant)

Some participants expressed motivation to dedicate more time to this type of universal prevention work that would reach all students, rather than becoming completely absorbed by the needs of individual students:

…I think it’s much more useful than sitting endlessly in one-on-one conversation. (interview, school nurse)

However, they reported an increasing demand from students for one-to-one conversations, which left them feeling conflicted:

It should be easy to get in touch… when you are out [of your office] in a meeting or you are teaching … the students come to a closed door. (FGD)

One participant did not agree that responsibility for critical health literacy should fall on school nurses, but on teachers:

…Contact the Directorate of Education…[include in] life skills subjects…. (interview, government consultant)

### Capability

Classroom teaching is already a part of what Norwegian school nurses are expected to be able to do. While some may have the capability to teach critical health literacy, many will need some form of support, either through structured learning resources that are easy to use without much prior knowledge or through training modules.

#### IHC Key Concepts are somewhat familiar but do not necessarily represent active knowledge

Participants expressed familiarity with some of the IHC Key Concepts as they resembled evidence-based principles they were exposed to during nursing studies. But this type of knowledge did not play an important role in their current practice:

You have to fend for yourself after the education, the evidence-based way of thinking quickly fades…. (FGD)

It is foreign… [we are] very practice-oriented and practical all the time. (FGD)

#### School nurses have varying teaching skills

Most participants had teaching experience. However, they pointed out that younger nurses may have more teacher training and feel more comfortable with classroom teaching and digital technology than older nurses.

Several valued teacher presence in the classroom, bringing pedagogical expertise and knowledge of the students:

We prefer that they are in the classroom when we have lessons. They know the students. We don't know them very well. (FDG)

### Opportunity

There are limited opportunities for Norwegian school nurses to teach critical health literacy due to a full workload largely determined by the ‘strong recommendations’ in the national practice guidelines. Collaboration with teachers and schools was desirable and seen as necessary for finding teaching opportunities. Participants also expressed the need for better collaboration at higher levels of governance.

#### Little time for ‘optional’ tasks

School nurses in Norway likely have very limited opportunities for teaching critical health literacy. Despite consensus about the seriousness of the problem, participants reported that since such teaching was not explicitly spelled out in the national guidelines, it would not likely be prioritized. Few had time for any tasks other than ‘strong recommendations’ found in the guidelines, though nurses working in schools with better nurse-to-student ratio had more flexibility for optional tasks.

If it feels like an extra thing to do, then I think maybe the door will shut quite quickly. (interview, researcher/school nurse)

…[we are] locked in that one-to-one conversation … a growing problem during and after the pandemic… universal interventions like education, for example, that’s what we down-prioritize completely. (interview, school nurse)

Some suggested incorporating critical health literacy teaching into existing responsibilities, such as fifth-sixth grade sexual health education or substituting some of the time set aside for eighth grade individual conversations.

#### School collaboration varies and is not established at a systems level

As the Norwegian school curriculum emphasizes critical thinking and ‘public health and life skills’ as cross-cutting topics, we explored participants’ views on collaborating with teachers in order to reach students.

Participants suggested several school subjects that could be relevant, such as physical activity, math, and science, or for interdisciplinary themed days. However, this would entail close collaboration with teachers, something that currently varied:

We collaborate very, very closely with the teachers. (FDG

…we do very little together with the teachers…. (FDG)

Few had collaborated with teachers on lesson planning.

We learned that any collaboration with schools must be negotiated locally, either at the municipality or school level, due to a lack of system-level collaboration between the educational system and school health services. The school health services are obliged to collaborate with their schools, but not necessarily vice versa. Collaboration was desirable and seen as important to facilitate any classroom teaching time. Some suggested advocacy activities:

We need to spend more time on the system, and then we can get involved. Speak at the principal’s meeting or talk to the school management. (interview, school nurse/project manager)

#### Other teaching opportunities

Participants suggested alternative populations and settings, including teachers’ meetings, parent–school meetings, or at health stations to reach pregnant mothers.

### Findings related to resource development

There was a broad consensus among all participants that teaching resources were lacking and would be valued. After much discussion about age relevance, workshop participants recommended that initial resources should be tailored to learners aged 10 and agreed on eight IHC Key Concepts for this group. See Table 4 [with concept numbers corresponding to IHC Key Concepts final 2022 version (Oxman *et al*. 2022) and links to explanations on IHC web site].

**Table 4:** IHC Key Concepts prioritized for students age 10.

Main category	Key concept	Short title w/ link to explanation
Claims	1.1e: Do not assume that comparisons are not needed	Messages with no comparison
	1.1a: Do not assume that treatments are safe	Messages that ignore harms
	1.3c: Do not assume that a treatment is helpful or safe based on how widely used it is or has been	Belief that commonly used means effective
	1.3d: Do not assume that a treatment is better based on how new or technologically impressive it is	Belief that new is better
	1.4a: Do not assume that personal experiences alone are sufficient	Trust in personal experiences
Comparisons	2.1a: Consider whether the people being compared were similar	Similar comparison groups
	2.4a: Be cautious of small studies	Small studies
Choices	3.2a: Weigh the benefits and savings against the harms and costs of acting or not	Benefits and harms

Findings related to resource needs are articulated in Table 5 using the format ‘User needs X so that they can Y’. This format can help resource developers in the next phase focus on user goals, rather than dictating solutions to them. It also provides a possible metrics for assessing success.

**Table 5:** School nurses’ needs related to resources.

School nurses need…	So that they can…	Quotes
…quality-assessed resources from a centralized source	Save time Be confident Provide consistent teaching regardless of location	‘Need a [centralized] portal of some kind’ (Interview 2) ‘Very important to have a good plan for evaluating’ (Interview 1)
…tailorable resources (‘skeleton’)	Reach different audiences (ages, genders) Incorporate trending topics	‘Huge differences… They don’t want the same topics and type of information’ (Interview 4)
…resources for short sessions that can be tacked on to existing teaching	Identify teaching opportunities	‘Getting access [to schools/students] I think will be a big barrier.’ ‘Put it into existing [teaching] would be smart’ (Interview 2)
…resources with supportive features and/or training modules	Use resources easily even with less content knowledge or teaching experience	‘It’s not only students that can’t critically assess claims but also nurses with a bachelor’s degree’ (Interview 2)
…resources that are aligned with national guidelines	Avoid burden due to extra workload Feel supported in carrying out mandated tasks	‘Should function as a resource for school health services’ mandates to carry out guidelines’ (Interview 2)
…resources that aligned with school curricular goals	Demonstrate value for school Identify teaching opportunities Facilitate collaboration	‘Many [people] want to get things into the schools’ (Interview 6) ‘You have support learning goals of the school, to help with what they are already doing’ (Interview 2)
…materials to present to others (municipalities, school leadership, teachers, and parents)	Facilitate collaboration Reinforce learning across school/family	‘Very important: support from [school] leadership’ *FGD* ‘This is important that parents know—could use in parents meetings’ (FGD)
Resources that engage students	Facilitate learning	‘Reach the whole class… trigger reflection and discussion’ (Interview 5) Preferably video FGD notes

## Discussion

We conducted this qualitative context analysis to explore key issues related to Norwegian school nurses’ motivation, capability, and opportunity to teach critical health literacy and to investigate possible needs in order to inform resource design in subsequent work. This study is part of a larger project to develop and test the effect of school nurse teaching resources on students’ critical health literacy.

### Strengths and limitations

Our scope was narrow, but we argue that the method is best suited to our inquiry of interest—to identify the most salient issues that can guide the future design and implementation of tailored teaching resources for school nurses. This aim differs somewhat from a more traditional qualitative study (Suchman *et al*. 2023) and may have led us to overlook issues not relevant to developing resources. Our long-term engagement in work related to the IHC Key Concepts may have introduced a bias in our questioning and analysis. We took measures to counteract this by continually assuring participants that we particularly valued responses that took opposing viewpoints or that challenged underlying assumptions about the value of increasing critical health literacy more generally. We also actively looked for varying viewpoints in the analysis. While it is a limitation that all participants are female, this is also fairly representative of school nurses in Norway.

The strength of this study includes multiple methods of inquiry, data collection from the perspective of different actors from the school health services sector, and multiple researchers analysing the data. While a larger sample might have uncovered a broader range of perspectives, our findings suggest that there is considerable consensus on key issues relevant to resource design, such as the likelihood of school nurses being motivated to use resources due to their experience of students’ vulnerability to misinformation, their need for solutions that can be easily used by school nurses with less critical health literacy skill and that can be tailored to include different health topics, and challenges related to limited teaching opportunities.

### Schools are an important arena for improving critical health literacy

Enacting policies to develop a population’s critical health literacy may have long-reaching societal impacts by building resilience to harmful information and improving people’s decision-making skills, which in turn can improve their health and well-being. Policies focused on strengthening health literacy more generally may also contribute to reducing health inequities, as differences in health literacy have a key role in explaining health disparities (de Buhr *et al*. 2020). Additionally, advancing people’s skills to think critically about evidence and make informed decisions can lead to thinking critically about interventions in other domains, such as climate change or social programmes (Aronson *et al*. 2019), and foster an informed, participatory citizenry. Schools represent an ideal context to teach these skills in settings such as Norway where most children attend school. However, critical health literacy is missing from K-12 education in many countries (Finland being a notable exception). A WHO report states:

Health literacy, the ability of individuals to understand, critically appraise and use information related to their health, is an important component of education and has become more prominent during the COVID-19 pandemic. Approaches to improving health literacy education in schools are lacking in many European countries. (World Health Organization 2021)

School nurses represent a potential pathway to strengthening critical health literacy skills. Often highly trusted as public health communicators, they are in a unique position to reach a large segment of the population at a young age and help young people and their families interpret public health information (Nash *et al*. 2021, Hoffman *et al*. 2024, Kühne and Mugo 2025), thereby potentially strengthening their resilience to health misinformation. However, they have competing commitments. In a systematic overview of overviews, authors classify school nurse’s working fields according to three core aspects of school health: (i) health literacy, (ii) medical healthcare, and (iii) health promotion (Pawils *et al*. 2023). School nurses in our study described the challenge of balancing these core responsibilities. Their roles may need to be reassessed in line with the public health threat of health misinformation.

An additional challenge is that they may lack skills and confidence related to their own critical health literacy. The findings from the current study are in line with previous studies that have shown that nurses, including public health nurses, may be reluctant to search for research themselves, citing weak appraisal skills and beliefs about the limitations of their role (Austvoll-Dahlgren and Helseth 2012, Hietaniemi *et al*. 2026) Their capacity and confidence to practice and promote critical health literacy should be increased (Hoekstra *et al*. 2016, de Buhr *et al*. 2020).

Collaboration with schools is central to implementation (Kostenius 2023). Other studies have called for interprofessional collaboration in schools and the need for pedagogical tools (Ringsberg *et al*. 2018), as well as the need for school nurses to initiate communication with school personnel (Nygård *et al*. 2024). However, a Norwegian survey from 2023 supports our finding concerning time constraints: school nurses in Norway are overworked, largely due to understaffing relative to demand (Landsgruppen av helsesykepleiere NSF 2023), and health prevention through teaching often takes a backseat to individual student needs and drop-in consultations.

Collaboration between schools and school health services varies and can be challenging for individual school nurses to negotiate (Helleve *et al*. 2022, Bråten 2024). Strengthening knowledge and skills to support resilience among young people to the emerging threat of misinformation should not fall on individual school nurses, but be facilitated through education and public health authorities’ collaboration at a systems level. Providing well-designed, quality-controlled resources through a free national distribution channel can be one part of a systematic approach.

## Conclusion

Norwegian school nurses are likely motivated and—with supportive resources—likely capable of teaching critical health literacy as a means of supporting resilience to misinformation among young people. Yet teaching opportunities remain scarce. Future resources should account for the limitations and challenges identified in this study: namely they should be clearly aligned with current school and health service goals, facilitate collaboration, require minimal class time, and be easy to access and use while engaging and activating students. Teaching should start early and evolve with students’ needs and topic trends. Future research should explore student perspectives when developing resources, including those in marginalized groups, and evaluate the resources’ effectiveness on students’ critical health literacy knowledge, skills, and competencies.

## Supplementary Material

daag083_Supplementary_Data

## Data Availability

The data is anonymized, but due to the small national setting, there is potential for identifying participants. Therefore, the data underlying this article will be shared on reasonable request to the corresponding author.

## References

[daag083-B1] Aronson JK, Barends E, Boruch R et al Key concepts for making informed choices. Nature 2019;572:303–6. 10.1038/d41586-019-02407-9 31406318

[daag083-B2] Austvoll-Dahlgren A, Guttersrud Ø, Nsangi A et al Measuring ability to assess claims about treatment effects: a latent trait analysis of items from the ‘Claim Evaluation Tools’ database using Rasch modelling. BMJ Open 2017;7:e013185. 10.1136/bmjopen-2016-013185 PMC5777469 28550019

[daag083-B3] Austvoll-Dahlgren A, Helseth S. Public health nurses’ barriers and facilitators to the use of research in consultations about childhood vaccinations. Scand J Caring Sci 2012;26:271–8. 10.1111/j.1471-6712.2011.00928.x 22171572

[daag083-B4] Austvoll-Dahlgren A, Oxman AD, Chalmers I et al Key concepts that people need to understand to assess claims about treatment effects. J Evid Based Med 2015;8:112–25. 10.1111/jebm.12160 26107552

[daag083-B5] Bråten AF . Tverrfaglig samarbeid mellom skole og skolehelsetjeneste. En analyse av nasjonale styringsdokumenter. Norway: Oslo Metropolitan University, 2024.

[daag083-B6] Chalmers I, Oxman AD, Austvoll-Dahlgren A et al Key Concepts for Informed Health Choices: a framework for helping people learn how to assess treatment claims and make informed choices. BMJ Evid Based Med 2018;23:29–33. 10.1136/ebmed-2017-110829 29367324

[daag083-B7] Chesire F, Mugisha M, Ssenyonga R et al Effects of the Informed Health Choices secondary school intervention: a prospective meta-analysis. J Evid Based Med 2023;16:321–31. 10.1111/jebm.12552 37735807

[daag083-B8] Chesire F, Mugisha M, Ssenyonga R et al Effects of the informed health choices secondary school intervention after 1 year: a prospective meta-analysis using individual participant data. Trials 2024a;25:733. 10.1186/s13063-024-08577-w 39478569 PMC11523815

[daag083-B9] Chesire F, Ochieng M, Mugisha M et al Contextualizing critical thinking about health using digital technology in secondary schools in Kenya: a qualitative analysis. Pilot Feasibility Stud 2022;8:227. 10.1186/s40814-022-01183-0 36203201 PMC9535840

[daag083-B10] Chesire F, Oxman AD, Kaseje M et al Process evaluation of teaching critical thinking about health using the Informed Health Choices Intervention in Kenya: a mixed methods study. Glob Health Sci Pract 2024b;12:2300485. 10.9745/GHSP-D-23-00485 PMC11666096 39706679

[daag083-B11] Chinn D . Critical health literacy: a review and critical analysis. Soc Sci Med 2011;73:60–7. 10.1016/j.socscimed.2011.04.004 21640456

[daag083-B12] Dahlgren A, Furuseth-Olsen K, Rose CJ et al The Norwegian public’s ability to assess treatment claims: results of a cross-sectional study of critical health literacy. F1000Research 2021;9:179. 10.12688/f1000research.21902.2 38585673 PMC10995534

[daag083-B13] de Buhr E, Ewers M, Tannen A. Potentials of school nursing for strengthening the health literacy of children, parents and teachers. Int J Environ Res Public Health 2020;17:2577. 10.3390/ijerph17072577 32283733 PMC7178108

[daag083-B14] Denniss E, Lindberg R. Social media and the spread of misinformation: infectious and a threat to public health. Health Promot Int 2025;40:daaf023. 10.1093/heapro/daaf023 40159949 PMC11955583

[daag083-B15] Do Nascimento IJB, Pizarro AB, Almeida JM et al Infodemics and health misinformation: a systematic review of reviews. Bull World Health Organ 2022;100:544–61. 10.2471/BLT.21.287654 36062247 PMC9421549

[daag083-B16] Ennis RH, Ennis SF. *What is critical thinking? 2003–2024*. https://criticalthinking.net/what-is-critical-thinking/. (22 June 2026, date last accessed).

[daag083-B17] Göttgens I, Oertelt-Prigione S. The application of human-centered design approaches in health research and innovation: a narrative review of current practices. JMIR Mhealth Uhealth 2021;9:e28102. 10.2196/28102 34874893 PMC8691403

[daag083-B18] Haugen ALH, Riiser K, Esser-Noethlichs M et al Fostering pupils’ critical health literacy: examining the potential of physical education in lower secondary school. Front Sports Act Living 2023;5:1205716. 10.3389/fspor.2023.1205716 37383063 PMC10294679

[daag083-B19] Helleve A, Midthassel UV, Federici RA. Finding the balance between collaboration and autonomy among school nurses in interactions with schools. J Sch Nurs 2022;38:184–93. 10.1177/1059840520918924 32308104 PMC8907552

[daag083-B20] Hietaniemi AM, Ylimäki S, Härkönen H et al Nurses’ experiences and perceptions of evidence-based healthcare competence: a qualitative systematic review. J Adv Nurs 2026;82:7083-7103. 10.1111/jan.70399 PMC13267469 41267476

[daag083-B21] Hoekstra BA, Young VL, Eley CV et al School nurses’ perspectives on the role of the school nurse in health education and health promotion in England: a qualitative study. BMC Nurs 2016;15:73. 10.1186/s12912-016-0194-y 28050164 PMC5203702

[daag083-B22] Hoffman R, Kay SS, Piepenbrink RP et al *Engaging School Communities During COVID-19: The Role of School Nurses*. American Journal of Public Health 2024:114:S402-S404. 10.2105/AJPH.2024.307591 PMC11111382 38547464

[daag083-B23] Kbaier D, Kane A, McJury M et al Prevalence of health misinformation on social media—challenges and mitigation before, during, and beyond the COVID-19 pandemic: scoping literature review. J Med Internet Res 2024;26:e38786. 10.2196/38786 39159456 PMC11369541

[daag083-B24] Kostenius C . School nurses’ experiences with health dialogues: a Swedish case. J Sch Nurs 2023;39:345–56. 10.1177/10598405211022597 34155940 PMC10486162

[daag083-B25] Kühne L, Mugo F. Investigating primary school nurses’ activities that are effective in health promotion and primary prevention: a systematic review. J Sch Health 2025;95:649–67. 10.1111/josh.70027 40566799 PMC12241768

[daag083-B26] Landsgruppen av helsesykepleiere NSF . Rapport: Helsesykepleiernes arbeidshverdag. 2023. Rapport 1:2023. https://actis.no/images/Rapport_Helsesykepleiere_endelig-versjon-komprimert-for-nett.pdf .

[daag083-B27] Michie S, Van Stralen MM, West R. The behaviour change wheel: a new method for characterising and designing behaviour change interventions. Implement Sci 2011;6:42. 10.1186/1748-5908-6-42 21513547 PMC3096582

[daag083-B28] Mugisha M, Oxman AD, Nyirazinyoye L et al Process evaluation of teaching critical thinking about health using the Informed Health Choices intervention in Rwanda: a mixed methods study. Glob Health Sci Pract 2024;12:e2300483. 10.9745/GHSP-D-23-00483 39706678 PMC11666086

[daag083-B29] Mugisha M, Uwitonze AM, Chesire F et al Teaching critical thinking about health using digital technology in lower secondary schools in Rwanda: a qualitative context analysis. PLoS One 2021;16:e0248773. 10.1371/journal.pone.0248773 33750971 PMC7984628

[daag083-B30] Nash R, Patterson K, Flittner A et al School-based health literacy programs for children (2–16 Years): an international review. J Sch Health 2021;91:632–49. 10.1111/josh.13054 34096058

[daag083-B31] Newman N, Fletcher R, Kalogeropoulos A et al Reuters institute digital news report 2019 (p. 156). Retrieved from Reuters Institute for the Study of Journalism. 2019. https://reutersinstitute.politics.ox.ac.uk/sites/default/files/2019-06/DNR_2019_FINAL_0.pdf

[daag083-B32] Nordheim LV, Pettersen KS, Espehaug B et al Lower secondary school students’ scientific literacy and their proficiency in identifying and appraising health claims in news media: a secondary analysis using large-scale survey data. BMJ Open 2019;9:e028781. 10.1136/bmjopen-2018-028781 PMC6803159 31630100

[daag083-B33] NRK . Unge kvinner dropper hormonell prevensjon etter råd på TikTok – samtidig øker aborttallene i Oslo. 2024. https://www.nrk.no/stor-oslo/unge-kvinner-dropper-hormonell-prevensjon-etter-rad-pa-tiktok–samtidig-oker-aborttallene-i-oslo-1.16649862

[daag083-B34] Nsangi A, Semakula D, Glenton C et al Informed health choices intervention to teach primary school children in low-income countries to assess claims about treatment effects: process evaluation. BMJ Open 2019;9:e030787. 10.1136/bmjopen-2019-030787 PMC6747654 31511291

[daag083-B35] Nsangi A, Semakula D, Oxman AD et al Effects of the Informed Health Choices primary school intervention on the ability of children in Uganda to assess the reliability of claims about treatment effects: a cluster-randomised controlled trial. The Lancet 2017;390:374–88. 10.1016/S0140-6736(17)31226-6 28539194

[daag083-B36] Nsangi A, Semakula D, Oxman AD et al Effects of the Informed Health Choices primary school intervention on the ability of children in Uganda to assess the reliability of claims about treatment effects, 1-year follow-up: a cluster-randomised trial. Trials 2020a;21:27. 10.1186/s13063-019-3960-9 31907013 PMC6945419

[daag083-B37] Nsangi A, Semakula D, Rosenbaum SE et al Development of the informed health choices resources in four countries to teach primary school children to assess claims about treatment effects: a qualitative study employing a user-centred approach. Pilot Feasibility Stud 2020b;6:18. 10.1186/s40814-020-00565-6 32055405 PMC7008535

[daag083-B38] Nutbeam D . The evolving concept of health literacy. Soc Sci Med 2008;67:2072–8. 10.1016/j.socscimed.2008.09.050 18952344

[daag083-B39] Nygård IJ, Hallström IK, Sollesnes R. Norwegian Public Health Nurses' Perspectives on Their Role in High Schools-A Qualitative Study. *Public Health Nursing* 2025;42:723–733. 10.1111/phn.13475 39690732

[daag083-B40] Oxman A, Chalmers I, Austvoll-Dahlgren A et al Key Concepts for assessing claims about treatment effects and making well-informed treatment choices [version 2; peer review: 3 approved]. F1000Research 2019;7:1784. 10.12688/f1000research.16771.2 PMC6290969 30631443

[daag083-B41] Oxman A, Chalmers I, Dahlgren A et al Key Concepts for assessing claims about treatment effects and making well-informed treatment choices (Version 2022). IHC Working Paper. 2022. Zenodo. 10.5281/zenodo.6611932 PMC6290969 30631443

[daag083-B42] Oxman AD, García LM. Comparison of the Informed Health Choices Key Concepts Framework to other frameworks relevant to teaching and learning how to think critically about health claims and choices: a systematic review. F1000Research 2020;9:164. 10.12688/f1000research.21858.1 33224475 PMC7670481

[daag083-B43] Oxman M, Rosenbaum S, Nsangi A et al The Health Choices Book: Learning to think carefully about treatments. A health science book for primary school children. 2016. http://www.informedhealthchoices.org/primary-school-resources/10.1177/0025817218755850 29380666

[daag083-B44] Pawils S, Heumann S, Schneider SA et al The current state of international research on the effectiveness of school nurses in promoting the health of children and adolescents: an overview of reviews. PLoS One 2023;18:e0275724. 10.1371/journal.pone.0275724 36812235 PMC9946271

[daag083-B45] Pedersen C, Wollscheid S. Samarbeid mellom skole og skolehelsetjeneste–hva forskningen forteller oss. *Bedre skole-tidskrift for lærere og skoleledere, 3/*2023. 2024.

[daag083-B46] Riiser K, Haugen ALH, Helseth S et al The Norwegian perspective. In: Global Perspectives on Children's Health Literacy: Intersections Between Health, Education and Community. Cham: Springer International Publishing, 2024, 175–89

[daag083-B47] Ringsberg KC, Olander E, Tillgren P et al Concerns and future challenges of health literacy in the Nordic countries–from the point of view of health promotion practitioners and researchers. Scand J Public Health 2018;46:107–17. 10.1177/1403494817743903 29552970

[daag083-B48] Rosenbaum S, Moberg J, Chesire F et al Teaching critical thinking about health information and choices in secondary schools: human-centred design of digital resources. F1000Research 2024;12:481. 10.12688/f1000research.132580.2 39246586 PMC11377934

[daag083-B49] Scherer LD, McPhetres J, Pennycook G et al Who is susceptible to online health misinformation? A test of four psychosocial hypotheses. Health Psychol 2021;40:274–84. 10.1037/hea0000978 33646806

[daag083-B50] Smith SK, Nutbeam D, McCaffery KJ. Insights into the concept and measurement of health literacy from a study of shared decision-making in a low literacy population. J Health Psychol 2013;18:1011–22. 10.1177/1359105312468192 23676466

[daag083-B51] Sørensen K, Van den Broucke S, Fullam J et al Health literacy and public health: a systematic review and integration of definitions and models. BMC Public Health 2012;12:80. 10.1186/1471-2458-12-80 22276600 PMC3292515

[daag083-B52] Ssenyonga R, Lewin S, Nakyejwe E et al Process evaluation of teaching critical thinking about health using the Informed Health Choices Intervention in Uganda: a mixed methods study. Glob Health Sci Pract 2024;12:e2300484. 10.9745/GHSP-D-23-00484 39706681 PMC11666090

[daag083-B53] Ssenyonga R, Sewankambo NK, Mugagga SK et al Learning to think critically about health using digital technology in Ugandan lower secondary schools: a contextual analysis. PLoS One 2022;17:e0260367. 10.1371/journal.pone.0260367 35108268 PMC8809610

[daag083-B54] Suchman L, Omoluabi E, Kramer J et al Analyzing fast and slow: combining traditional and rapid qualitative analysis to meet multiple objectives of a complex transnational study. Front Sociol 2023;8:961202. 10.3389/fsoc.2023.961202 36818663 PMC9931144

[daag083-B55] Tong A, Sainsbury P, Craig J. Consolidated criteria for reporting qualitative research (COREQ): a 32-item checklist for interviews and focus groups. Int J Qual Health Care 2007;19:349–57. 10.1093/intqhc/mzm042 17872937

[daag083-B56] Vosoughi S, Roy D, Aral S. The spread of true and false news online. Science 2018;359:1146–51. 10.1126/science.aap9559 29590045

[daag083-B57] Wilson M, Vélez M, Lavis J. Impact of strategies to mitigate misinformation in diverse settings and populations: a protocol for a living evidence synthesis. BMJ Open 2023;13:e076672. 10.1136/bmjopen-2023-076672 PMC10582920 37827737

[daag083-B58] World Health Organization . Health Literacy in the Context of Health, Well-Being and Learning Outcomes the Case of Children and Adolescents in Schools: the Case of Children and Adolescents in Schools (No. WHO/EURO: 2021-2846-42604-59268). World Health Organization. Regional Office for Europe, 2021.

[daag083-B59] Zhou L . *The danger of Meta’s big fact-checking changes*. 2025. https://www.vox.com/politics/393863/meta-mark-zuckerberg-fact-checking-trump

