# Supplementary material for: Exploring school nurses’ potential to strengthen young people’s resilience to misinformation by promoting critical health literacy in Norway
Source: Health Promot Int. 2026 Jun 26;41(3):daag083. doi: 10.1093/heapro/daag083 (PMC13308653; doi:10.1093/heapro/daag083)
Supplement: daag083_Supplementary_Data [file daag083_supplementary_data.zip › Supplementary file 2.doc]

# Supplementary file 2. COREQ checklist

**Consolidated criteria for reporting qualitative studies (COREQ): 32-item checklist**

Please indicate in which section each item has been reported in your manuscript. If you do not feel an item applies to your manuscript, please enter N/A.

For further information about the COREQ guidelines, please see Tong *et al.*, 2017:

https://doi.org/10.1093/intqhc/mzm042

| **No.** | **Item** | **Description** | **Section(s)** | **Page#** |
| --- | --- | --- | --- | --- |
| **Domain 1: Research team and reflexivity** | | |  |  |
| Personal characteristics | | |  |  |
| *1.* | Interviewer/facilitator | Which author/s conducted the interview or focus group? | - Author contributions  - Methods/Data collection/  - Semi-structured interviews  - Focus group discussions  - Co-design workshop | 19 |
| *2.* | Credentials | What were the researcher's credentials? *E.g. PhD, MD* | - Author contributions | 19 |
| *3.* | Occupation | What was their occupation at the time of the study? | - Author contributions | 19 |
| *4.* | Gender | Was the researcher male or female? | Methods/Reflexivity | 10 |
| *5.* | Experience and training | What experience or training did the researcher have? | Methods/Data collection | 8 |
| Relationship with participants | | |  |  |
| *6.* | Relationship established | Was a relationship established prior to study commencement? | Table 2. Participant selection, recruitment, compensation | 7-8 |
| *7.* | Participant knowledge of the interviewer | What did the participants know about the researcher? *E.g. Personal goals, reasons for doing the research* | What was communicated is included in the interview/fgd guides. However, these are not submitted as Supplementary files since they are in norwegian. Can be provided on request from corresponding author. | See comment in previous column |
| *8.* | Interviewer characteristics | What characteristics were reported about the interviewer/facilitator? *E.g. Bias, assumptions, reasons and interests in the research topic* | - Methods/Reflexivity - Discussion/Strengths and limitations | 10  17 |
| **Domain 2: Study design** | | |  |  |
| Theoretical framework | | |  |  |
| *9.* | Methodological orientation and theory | What methodological orientation was stated to underpin the study? *E.g. grounded theory, discourse analysis, ethnography, phenomenology, content analysis* | - Methods (first paragraph) | 6 |
| Participant selection | | |  |  |
| *10.* | Sampling | How were participants selected? *E.g. purposive, convenience, consecutive, snowball* | Methods/   - Participation selection and recruitment - Table 2. | 7-8 |
| *11.* | Method of approach | How were participants approached? *E.g. faceto-face, telephone, mail, email* | Methods/   - Participation selection and recruitment - Table 2. | 7-8 |
| *12.* | Sample size | How many participants were in the study? | - Results (first paragraph) - Table 3 | 10 |
| *13.* | Non-participation | How many people refused to participate or dropped out? What were the reasons for this? | n/a | n/a |
| Setting | | |  |  |
| *14.* | Setting of data collection | Where was the data collected? *E.g. home, clinic, workplace* | Methods/Data collection   - Semi-structured interviews - Focus group discussions - Workshop | 6-7 |
| *15.* | Presence of nonparticipants | Was anyone else present besides the participants and researchers? | n/a | n/a |
| *16.* | Description of sample | What are the important characteristics of the sample? *E.g. demographic data, date* | Results (first paragraph) and  Table 3 | 10 |
| Data collection | | | |  |
| *17.* | Interview guide | Were questions, prompts, guides provided by the authors? Was it pilot tested? | Methods/Data collection/   - Semi structured interviews - Focus group discussions - Co-design workshop   Appendix 4 | 8 |
| *18.* | Repeat interviews | Were repeat interviews carried out? If yes, how many? | n/a | n/a |
| *19.* | Audio/visual recording | Did the research use audio or visual recording to collect the data? | Methods/Data collection/   - Semi structured interviews - Focus group discussions - Co-design workshop | 8 |
| *20.* | Field notes | Were field notes made during and/or after the interview or focus group? | Methods/Data collection/   - Semi structured interviews - Focus group discussion - Co-design workshop | 8 |
| *21.* | Duration | What was the duration of the interviews or focus group? | Methods/Data collection/   - Semi structured interviews - Focus group discussion - Co-design workshop | 8 |
| *22.* | Data saturation | Was data saturation discussed? | Discussion/Strengths and limitations | 17 |
| *23.* | Transcripts returned | Were transcripts returned to participants for comment and/or correction? | no | - |
| **Domain 3: analysis and findings** | | | |  |
| Data analysis | | | |  |
| *24.* | Number of data coders | How many data coders coded the data? | Methods/Data analysis/   - Interviews and FGDs - Co-design workshop | 9 |
| *25.* | Description of the coding tree | Did authors provide a description of the coding tree? | - Supplementary file 4 | Supplementary File 4 |
| *26.* | Derivation of themes | Were themes identified in advance or derived from the data? | Methods/Data analysis/   - Interviews and FGDs - Co-design workshop | 9 |
| *27.* | Software | What software, if applicable, was used to manage the data? | Methods/Data analysis/   - Interviews and FGDs | 9 |
| *28.* | Participant checking | Did participants provide feedback on the findings? | no | - |
| Reporting | | | |  |
| *29.* | Quotations presented | Were participant quotations presented to illustrate the themes / findings? Was each quotation identified? *E.g. Participant number* | Results | 10-16 |
| *30.* | Data and findings consistent | Was there consistency between the data presented and the findings? | Results | 10-16 |
| *31.* | Clarity of major themes | Were major themes clearly presented in the findings? | Results | 10-16 |
| *32.* | Clarity of minor themes | Is there a description of diverse cases or discussion of minor themes? | Results | 10-16 |
