# Supplementary material for: Exploring school nurses’ potential to strengthen young people’s resilience to misinformation by promoting critical health literacy in Norway
Source: Health Promot Int. 2026 Jun 26;41(3):daag083. doi: 10.1093/heapro/daag083 (PMC13308653; doi:10.1093/heapro/daag083)
Supplement: daag083_Supplementary_Data [file daag083_supplementary_data.zip › Supplementary file 3.doc]

# Appendix 3. Document analysis details

## Methods detail

*Table 1. Included documents and selected parts for analysis*

|  | **Document title (english)** | **Dokument tittel/link (norsk)** | **The parts of the document we investigated:** |
| --- | --- | --- | --- |
| 1 | Regulations for Health Centres and School Health Services (2018 | [Forskrift om helsestasjons- og skolehelsetjenesten](https://lovdata.no/dokument/SF/forskrift/2018-10-19-1584) (2018) | Chapter 1,2,3,4 og 5, including sub-sections |
| 2 | Regulations for National Guidelines for Public Health Nurse Education (2021) | [Forskrift om nasjonal retningslinje for helsesykepleierutdanning](https://lovdata.no/dokument/SF/forskrift/2021-04-09-1146) (2021) | Chapter 1,2,3,4,5,6,7,8 og 9, including sub-sections. |
| 3 | National Professional Guidelines for Health Promotion and Preventive Work in Health Centers, School Health Services and Youth Health Centers (2017) | [Nasjonal faglig retningslinje for det helsefremmende og forebyggende arbeidet i helsestasjon, skolehelsetjenesten og helsestasjon for ungdom](https://www.helsedirektoratet.no/retningslinjer/helsestasjons-og-skolehelsetjenesten) (2017) | Chapter 1,2,3 (Shared core sections) and 5 (School Health Services 5-20 years) with sub-sections. |
| 4 | The curriculum for Master of Public Health Nursing, Western Norway University of Applied Sciences (HVL) | [Studieplanen for Master i helsesykepleie, Høgskulen på Vestlandet (HVL)](https://www.hvl.no/studier/studieprogram/helsesykepleie/2023h/studieplan/) | Program description and course descriptions for HEL510, HEL511,HEL512  HEL514, HEL515, HELP1, MAMET1HE  HEL590 |
| 5 | The curriculum for Master of Public Health Nursing, Molde University College (HiMolde) | [Studieplanen for Master i helsesykepleie, Høgskolen i Molde (HiMolde)](https://www.himolde.no/studier/programmer/mastergrad-i-helsesykepleie/studieplaner/2023.html) | Program description and course descriptions for MHP700, MHP730, MHP710, MHP740, MHP720, MHP750, MHP760, MHP770 |
| 6 | The curriculum for Master of Public Health Nursing, Inland University College (Hinn) | [Studieplanen for Master i helsesykepleie, Høgskolen i Innlandet (Hinn)](https://studiekatalog.edutorium.no/inn/nb/program/MAHEL/2023-host) | Program description and course descriptions for HEL4001, HEL4003, HEL4005, HEL4004, HEL4006, HEL4009 |
| 7 | The curriculum for Master of Public Health Nursing, NTNU (Trondheim) | [Studieplanen for Master i helsesykepleie, NTNU (Trondheim)](https://www.ntnu.no/studier/studieplan#programmeCode=MHELSP&year=2023&dir=MHELSP23-V-T) | Program description and the course descriptions for SYT3401, MH3002, SYT3403, SYT3402, SYT3406, SYT3404, SYT3405, MH3003, MH3004, SYT3408, SYT3904 |
| 8 | The curriculum for Master of Public Health Nursing, OsloMet | [Studieplanen for Master i helsesykepleie, OsloMet](https://student.oslomet.no/studier/-/studieinfo/programplan/MAHEL/2023/H%C3%98ST) | Program description and course descriptions for MAHEL4100, MAHEL4300, MAVIT4100, MAHELPRA10, MAVIT4050, MAVIT4060, MAVIT4070, MAVIT5910 |
| 9 | The curriculum for Master of Public Health Nursing, University of Agder (UiA) | [Studieplanen for Master i helsesykepleie, Universitetet i Agder (UiA)](https://www.uia.no/studier/program/helsesykepleie-master-2-ar/studieplaner/2023h.html) | Program description and course descriptions for SY-434-1, SY-435, SY-436-1, SY-437-1, SYP432-1, SY-438-1, SY-440-1, SY-439-1 |
| 10 | The curriculum for Master of Public Health Nursing, University of Stavanger (UiS) | [Studieplanen for Master i helsesykepleie, Universitetet i Stavanger (UiS)](https://www.uis.no/nb/studieprogram-og-emner/helsesykepleie-masterstudium/2023) | Program description and course descriptions for MHE100, MHE001, MHE110, MHE121, MHE130, MHEP40, MHE131, MHEPRO, |
| 11 | The curriculum for Master of Public Health Nursing, University of Tromsø (UiT) | [Studieplanen for Master i helsesykepleie, Universitetet i Tromsø (UiT)](https://uit.no/Content/757659/cache=20231409123654/Studieplan%20for%20M-HESYKPL%20Master%20i%20helsesykepleie%20kull%202022.pdf) | Program description and course descriptions for HEL-3151, HEL-3152, HEL-3167, HEL-3159, HEL-3160, HEL-3121, HEL-3161 |
| 12 | The curriculum for Master of Public Health Nursing, University of Southeast Norway (USN) | [Studieplanen for Master i helsesykepleie, Universitetet i Sørøst-Norge (USN)](https://www.usn.no/studier/studie-og-emneplaner/#/studieplan/MHELSE_2023_H%C3%98ST) | Program description and course descriptions for MHEL100, MHEL101, MHEL200, MHEL201, MHEL300, |
| 13 | Curriculum for continuing education in nursing, VID OSLO | [Studieplanen for videreutdanning i helsesykepleie, VID OSLO](https://www.vid.no/planer/studieplan-for-videreutdanning-i-helsesykepleie-2023-2024/) | Program description and course descriptions for MAHEL5010, MAHEL5040, MAHEL5030, MAHEL6500, MAHEL5400, MAHEL6200, MAHEL6900 |

**Notes about included documents**

In 2021, new regulations for public health nursing education came into effect, requiring implementation by autumn 2023 across educational institutions. We initially planned to extract data from the curricula for cohorts starting in autumn 2023. However, since some institutions do not admit new cohorts annually, we instead extracted the most recent curricula available on their websites.

**Excluded curricula and institutions**

We excluded descriptions of courses that focused exclusively on content related to infants and toddlers (0-5 years). Additionally, descriptions for a few courses were unavailable at the time of data extraction and are therefore not included in the study. At one institution - NTNU Ålesund - we did not find any available curricula when extracting data, consequently, the public health nursing program at NTNU Ålesund was not included in the dataset. The absence of a published curriculum in autumn 2023 is most likely due to the fact that new students are not admitted every year.

## Results detail

Table 2. Document analysis categories

| **“yes”** indicates text explicitly related to teaching critical health literacy | **"no"** indicates text without any reference to teaching critical health literacy. | **“indirect"** indicates text that can be linked indirectly to teaching critical health literacy, such as references to "health literacy", "promoting informed health choices", or similar concepts. | **"If yes/indirectly":**  Paste text that explicitly mentions teaching critical thinking about health claims or can be indirectly linked to this, such as references to health literacy pr promoting informed health choices. |
| --- | --- | --- | --- |

### Regulations for Health Centres and School Health Services (2018)

This document does not explicitly address teaching critical health literacy. Some sections are *indirectly* related, particularly those addressing health promotion in schools . According to the regulations, the school health service shall contribute to promoting physical and mental health among children and young people, for instance by strengthening their autonomy and skills to manage everyday lives and health-related conditions. Furthermore, the school health service is expected to provide information, support, and teaching in groups, classes, and at parent meetings as requested by the school. In addition, it shall contribute to the municipality's cross-sectoral public health work, including providing an overview of the health status and factors influencing the health of children and young people aged 0–20.

**Our interpretation of how the text can be indirectly linked to teaching critical health literacy in schools:** One factor influencing the health of children and young people is their health literacy, including their *critical* health literacy. By incorporating teaching on critical health literacy, the school health service, in collaboration with the school, can foster critical thinking about health claims and strengthen children’s and young people's ability to manage everyday life.

### National Professional Guideline for Health Promotion and Prevention Work in Health Centres, School Health Services and Youth Health Centres (2017)

This document does not explicitly address teaching critical health literacy, but some content is *indirectly* related. The guideline states that school health services should support schools in teaching curriculum topics and addressing challenges at the school or the local environment. Teaching should also cover topics raised in the school entrance examination and the Health Conversation in grade 8. It is strongly recommended that the service promote life skills and healthy lifestyle choices. Health Conversations should normalize common challenges and strengthen knowledge, attitudes and action skills. For grades 1 and 8, these conversations should foster dialogue with children, young people and parents about well-being, habits and health-related choices, while health personnel provide advice that supports healthy habits.

The guideline notes that it’s topics are not exhaustive, and encourages health personnel to address additional topics based on individual needs. It strongly recommends that school health services provide sexual health education and promote life skills that enable children and young people to take greater control of their health, thus making healthy choices easier. While the internet offers information on sexuality and boundaries, it may also contain misleading content; therefore, sex education by confident, knowledgeable adults is considered essential. The guideline also strongly recommends maintaining an overview of support materials for substance abuse prevention collaborating with schools to implement learning activities aligned with curriculum aims. These materials (e.g., Korusoslo.no, chapter 3) include objectives and activities that can directly or indirectly foster critical thinking about health claims.

**Our interpretation of how the text can be indirectly linked to teaching critical health literacy in schools:** The guideline states that the list of topics for teaching and Health Conversations is not exhaustive and may include issues such as information sources, media use, and critical thinking about health claims. Although it does not explicitly require teaching critical health literacy, there is a clear basis for it, as misleading or confusing health claims are among the challenges faced by children and young people. By supporting schools in this ares, the school health service can empower pupils to take greater control of their health and make informed choices.

### Regulations for National Guidelines for Public Health Nurse Education (2021):

This document does not explicitly address teaching critical health literacy, but some content is indirectly relevant. The regulations require public health nursing programs to meet specified learning outcomes, including knowledge of health literacy and the ability to use evidence-based methods for health promotion, prevention, and change management (chapters 2 to 7 for 120 credit programs and chapters 2 to 6 for 90-credit programs). Candidates should be able to promote informed health choices, coping skills and quality of life among children, young people, and their families/caregivers, apply knowledge of children and young people's digital platform use to implement appropriate measures. They must also understand how variations in health literacy affect disease management, self-care, and the ability to comprehend, evaluate and apply health information.

**Our interpretation of how the text can be indirectly linked to teaching critical health literacy in schools:** Although the regulations do not explicitly require preparing candidates (public health nurses) to teach critical health literacy, this competence can be inferred. Graduates are expected to have knowledge of health literacy, teaching, and children and young people's use of digital platforms, as well as skills to promote informed health choices. Accordingly, public health nurse candidates may be equipped to teach critical thinking about health claims.

### The study plans for Master of Public Health Nursing:

None of the Master of Public Health Nursing curricula explicitly address teaching critical health literacy, but some topics are indirectly related. The curricula emphasize knowledge of health literacy, methods for teaching health promotion, prevention, and change management, as well as understanding children’s and young people’s use of digital platforms and promoting informed health choices.

Many educational institutions follow the structure and formulations of the 2021 Regulations on National Guidelines for Public Health Nursing Education , though some do so to a lesser extent.

We used indirect formulations from the 2021 Regulations on National Guidelines for Public Health Nursing Education (Table 3) as a basis, as these may relate indirectly to teaching critical health literacy in schools. We marked with a cross whether similar formulations appear in the institutions’ curricula. For example, in chapter 1 (Scope and purpose) we selected the statement: “The candidate should be able to promote informed health choices”. If this wording was included in a program description, it was marked. Likewise, chapters 2-4 address learning outcomes within specific competence areas; similar formulations in course descriptions were also marked.

**Our interpretation of how the text can be indirectly linked to teaching critical health literacy in schools:** Although the curricula do not explicitly state that graduates should teach critical health literacy, they may still have developed this competence. The curricula emphasize knowledge of health literacy, methods for teaching health promotion, prevention, and change management, as well as understanding of children’s and young people's use of digital platforms. Such knowledge may enable candidates to teach critical health literacy.

*Table 3. Indirect references to critical health literacy from the 2021 Regulations on National Guidelines for Public Health Nursing Education identified in the Master of Public Health Nursing curricula:*

| **Chapter and paragraph** | HVL | Hi  Molde | HiNN | NTNU | Oslo  Met | UiA | UiS | UiT | USN | VID  OSLO |
| --- | --- | --- | --- | --- | --- | --- | --- | --- | --- | --- |
| **Chapter 1. Scope and purpose** | | | | | | | | | | |
| *§ 1 Purpose of the education*  -The candidate should be able to promote informed health choices | x | x | x | x |  | x |  | x |  |  |
| **Chapter 2. Learning outcomes - public health work** | | | | | | | | | | |
| *§ 4 Knowledge*  -has knowledge about health literacy and how variations in health literacy impact different user groups' disease management, coping, self-care, and their ability to understand, evaluate and apply health information | x | x | x |  | x |  | x | x |  | x |
| **Chapter 3. Learning outcomes - children, young people and their families/caregivers** | | | | | | | | | | |
| *§ 8 Skills*  -can apply evidence-based methods to promote informed health choices, coping skills, and quality of life in children, young people and their families/caregivers | x | x | x | x |  | x | x | x | x | x |
| *§ 8 Skills*  -can apply knowledge about children and young people's use of digital platforms to intervene when needed. | x | x | x | x | x |  | x | x |  | x |
| **Chapter 4. Learning outcomes - communication, relationships and interaction** | | | | | | | | | | |
| *§ 11 Skills*  -can use relevant methods to teach health promotion. prevention, and change management in an independent manner. | x | x |  | x | x | x | x | x | x | x |

**HVL**: Høgskulen på Vestlandet (Western Norway University of Applied Sciences). **Hi Molde**: Høgskolen i Molde (Molde University College). **HiNN**: Høgskolen i Innlandet (Inland Norway University of Applied Sciences, now University of Inland Norway). **NTNU**: Noregs teknisk-naturvitskaplege universitet (Norwegian University of Science and Technology). **OsloMet**: Oslo Metropolitan University. **UiA**: Universitet I Agder (University of Agder). **UiS**: Universitetet i Stavanger (University of Stavanger). **USN**: Universitetet i Sørøst-Norge (University of South-Eastern Norway. **VID Oslo**: VID vitenskapelige høgskole Oslo (VID Specialized University Oslo)

## What have we not looked at and why

Public health nursing programs are master's level and require a bachelor's degree in nursing for admission. Although we did not review the governing documents for the bachelor's degree, it is likely that public health nursing students enter programs with some knowledge of health literacy from their basic nursing education.
