# Supplementary material for: Exploring school nurses’ potential to strengthen young people’s resilience to misinformation by promoting critical health literacy in Norway
Source: Health Promot Int. 2026 Jun 26;41(3):daag083. doi: 10.1093/heapro/daag083 (PMC13308653; doi:10.1093/heapro/daag083)
Supplement: daag083_Supplementary_Data [file daag083_supplementary_data.zip › Supplementary file 4.doc]

# Supplementary file 4 Themes, sub-themes, codes

***Table 1: Initial themes and sub-themes, guided by findings from previous studies*** (Chesire et al., 2022), (Mugisha et al., 2021), (Ssenyonga et al., 2022).

*Capability, Opportunity, and Motivation refers to school nurses with regards to teaching IHC Key Concepts*.*

| **Top-level themes*** | **Sub-themes** | **Explanation** |
| --- | --- | --- |
| ***Capability*** | Role understanding and existing practice | Descriptions of how they work today that is effective (or not) |
|  | School nurse competence | Competence of school nurses regarding IHC topics |
|  | New resources - desirable characteristics | Expressed or implicit needs regarding learning resources - characteristics, type, format |
| ***Opportunity*** | Teaching opportunities/meeting points | Possible meeting points where learning can occur (students, teachers, parents) |
|  | Capacity, time | Time available, support from others, competing tasks |
|  | Collaboration with teachers and others | Teacher collaboration, support, and cooperation with others |
|  | Relevant topics | Health topics that are relevant and could be used as examples in teaching |
|  | How content is decided in their tasks | From document analysis or other sources |
| ***Motivation*** | Motivation/Perceived need for IHC resources | Motivation and perceived need among school nurses for IHC learning resources |
|  | Demand for school nurse teaching | Demand (from students/teachers/parents/others) for teaching by school nurses |
|  | Existing resources | Existing related learning resources in school health services/schools |

***Table 2: Final set of themes, sub-themes and codes***

*Capability, Opportunity, and Motivation refers to school nurses with regards to teaching IHC Key Concepts*.*

| **Top-level themes*** | **Sub-theme** | **Codes** |
| --- | --- | --- |
| ***Capability*** | Existing practice and role understanding | Who we (school nurses) are |
|  |  | What characterizes us and the service? |
|  |  | This is what healthcare nurses (we) do today |
|  | Ability and competence | Assumed core competence in the profession |
|  |  | Competence challenges |
|  |  | The vision for the service |
|  |  | Development of resources |
|  |  | Education of school nurse |
|  | Collaboration | Collaboration with other school nurses |
|  |  | Teachers / principal / school |
|  |  | Other collaboration |
|  | Department of Child and Adolescent Health Promotion Services (NASKO) | Expectations |
|  |  | Role |
| ***Opportunity*** | Existing practice and role understanding | Who we (school nurses) are. |
|  |  | What characterizes us and the service? |
|  |  | This is what healthcare nurses (we) do today |
|  |  | This is what we should do more of |
|  |  | Our challenges |
|  | Motivation and perceived need | The school's motivation |
|  | How is the content of school nurse's assignments decided | School nurse decides the scheme itself |
|  |  | The school management |
|  |  | Teaching must be agreed with the school |
|  |  | Political management and guidelines |
|  | School nurse's cooperation | Cooperation with others school nurse |
|  |  | Teachers / principal / school |
|  |  | Parents |
|  | Department of Child and Adolescent Health Promotion Services (NASKO) | Expectations |
|  |  | Role |
|  |  | Other cooperation |
|  | Teaching opportunities and meeting points | Existing meeting points between school nurse and students |
|  |  | Individual and cross-curricular school subjects / event days |
|  |  | Outside school hours |
|  |  | Be a backdrop everywhere |
|  | Target group | Age group |
|  |  | Age to start with IHC |
|  |  | Other target groups |
|  |  | Children and young people's encounter with social media |
|  |  | Finding out the needs of the target groups |
|  |  | The target groups have different starting points |
|  |  | Adaptation to the age group |
|  | Barriers | Assignment from above (incl. resources and implementation plans) |
|  |  | Uneven practice (equity?) |
|  |  | Coordination/system for sharing/ finding resources |
|  |  | Relationship with parents/guardians |
|  |  | Office vs teaching (see also important themes in Existing practice and understanding of roles) |
|  |  | Capacity/time |
|  |  | Competition with other resources/actors |
|  |  | Critical health skills take a long time time without visible results |
|  |  | How welcome school nurses are in school |
|  |  | Few guidelines for some age groups |
|  |  | Barriers in implementation and use of resources |
| ***Motivation*** | Existing practice and role understanding | Who we (school nurses) are. |
|  |  | What characterizes us and the service? |
|  |  | This is what school nurses do today |
|  |  | This is what we should do more of |
|  |  | Our (school nurses’) challenges |
|  | Motivation and perceived need | Children should learn this |
|  |  | Children's needs (from the perspective of the HSP/adults) |
|  |  | Health competence already important |
|  |  | School’s motivation |
|  | Barrier | Capacity/time |
|  |  | Critical health skills take a long time without visible results |
|  |  | How welcome school nurses are in school |
| ***New Resources: Desirable characteristics and ideas*****  ***We added this top-level theme as an expansion of one of the sub-theme from the initial framework “New resources - desirable characteristics”.* |  | Easy to find in a central place (findability) |
|  |  | Quality assured (credibiity) |
|  |  | Non-moralizing (desireability/identification) |
|  |  | Easy to use for all levels (usability – tailorability) |
|  |  | Theme mapping |
|  |  | Must involve children |
|  |  | Get parents involved |
|  |  | Ideas – products |
|  |  | Ideas – learning strategies and structure of teaching |
|  |  | Content must be linked to existing goals |
|  |  | Desired content, learning goals, quality |

Chesire, F., Ochieng, M., Mugisha, M., et al. (2022) Contextualizing critical thinking about health using digital technology in secondary schools in Kenya: a qualitative analysis. *Pilot and feasibility studies*, 8, 227. 10.1186/s40814-022-01183-0.

Mugisha, M., Uwitonze, A. M., Chesire, F., et al. (2021) Teaching critical thinking about health using digital technology in lower secondary schools in Rwanda: A qualitative context analysis. *PLoS One*, 16, e0248773.

Ssenyonga, R., Sewankambo, N. K., Mugagga, S. K., et al. (2022) Learning to think critically about health using digital technology in Ugandan lower secondary schools: A contextual analysis. *PLoS One*, 17, e0260367.
