# Supplementary material for: Exploring school nurses’ potential to strengthen young people’s resilience to misinformation by promoting critical health literacy in Norway
Source: Health Promot Int. 2026 Jun 26;41(3):daag083. doi: 10.1093/heapro/daag083 (PMC13308653; doi:10.1093/heapro/daag083)
Supplement: daag083_Supplementary_Data [file daag083_supplementary_data.zip › Supplementary file 1.doc]

| **1. Claims**  *Claims about effects that are not supported by evidence from fair comparisons are not necessarily wrong, but there is an insufficient basis for believing them.* | **2. Comparisons**  *To identify treatment effects, studies should make fair comparisons, designed to minimise the risk of systematic errors (biases) and random errors (the play of chance).* | **3. Choices**  *What to do depends on judgements about a problem, the relevance of the available evidence, and the balance of expected benefits, harms, and costs.* |
| --- | --- | --- |
| **1.1 Assumptions that treatments are safe or effective can be misleading.**  Do not assume that  a) treatments are safe,  b) treatments have large, dramatic  effects,  c) treatment effects are certain,  d) it is possible to know who will  benefit and who will be harmed, or  e) comparisons are not needed.  **1.2 Seemingly logical assumptions**  **about research can be misleading.**  Do not assume that  a) a plausible explanation is sufficient,  b) association is the same as causation,  c) more data is better data,  d) a single study is sufficient, or  e) fair comparisons are not applicable in practice.  **1.3 Seemingly logical assumptions**  **about treatments can be misleading.**  Do not assume that  a) treatment is needed,  b) more treatment is better,  c) a treatment is helpful or safe based  on how widely used it is or has been,  d) a treatment is better based on how  new or technologically impressive it  is, or  e) earlier detection of ‘disease’ is  better.  **1.4 Trust based on the source of a**  **claim alone can be misleading.**  Do not assume that  a) personal experiences alone are  sufficient,  b) your beliefs are correct,  c) opinions alone are sufficient,  d) peer review and publication is  sufficient, or  e) there are no competing interests. | **2.1 Comparisons of treatments**  **should be fair.**  Consider whether  a) the people being compared were  similar,  b) the people being compared were  cared for similarly,  c) the people being compared knew  which treatments they received,  d) outcomes were assessed similarly in  the people being compared,  e) outcomes were assessed reliably,  f) outcomes were assessed in all (or  nearly all) the people being  compared, and  g) people’s outcomes were analysed in  the group to which they were  allocated.  **2.2 Reviews of the effects of**  **treatments should be fair.**  Consider whether  a) systematic methods were used,  b) unpublished results were  considered,  c) treatments were compared across  studies, and  d) important assumptions were tested.  **2.3 Descriptions of effects should**  **clearly reflect the size of the effects.**  Be cautious of  a) verbal descriptions alone of the size  of effects,  b) relative effects of treatments alone,  c) average differences between  treatments, and  d) lack of evidence being interpreted as evidence of “no difference”.  **2.4 Descriptions of effects should**  **reflect the risk of being misled by the**  **play of chance.**  Be cautious of  a) small studies,  b) results for a selected group of people  within a study,  c) p-values, and  d) results reported as “statistically  significant” or “non-significant”. | **3.1 Evidence should be relevant.**  a) Be clear about what the problem or goal is and what the options are.  Consider the relevance of  b) the outcomes measured in the  research,  c) fair comparisons in laboratories,  animals, or highly selected people,  d) the treatments that were compared,  and  e) the circumstances in which the  treatments were compared.  **3.2 Expected advantages should**  **outweigh expected disadvantages.**  a) Weigh the benefits and savings  against the harms and costs of  acting or not.  Consider  b) the baseline risk or severity of the  symptoms when estimating the size  of expected effects,  c) how important each advantage and  disadvantage is when weighing the  pros and cons,  d) how certain you can be about each  advantage and disadvantage, and  e) the need for further fair  comparisons. |

www.informedhealthchoices.org
